# Tauroursodeoxycholic Acid Attenuates Renal Tubular Injury in a Mouse Model of Type 2 Diabetes

**DOI:** 10.3390/nu8100589

**Published:** 2016-09-22

**Authors:** Jing Zhang, Ying Fan, Chuchu Zeng, Li He, Niansong Wang

**Affiliations:** Department of Nephrology, Shanghai Jiao Tong University Affiliated Sixth People’s Hospital, Shanghai 200233, China; jingzhang721@163.com (J.Z.); fanyingsh@126.com (Y.F.); zengchuchu1991@yahoo.com (C.Z.); 12211280018@fudan.edu.cn (L.H.)

**Keywords:** diabetic nephropathy, endoplasmic reticulum stress, apoptosis, tauroursodeoxycholic acid

## Abstract

Renal tubular injury is a critical factor in the pathogenesis of diabetic nephropathy (DN). Endoplasmic reticulum (ER) stress is involved in diabetic nephropathy. Tauroursodeoxycholic acid (TUDCA) is an effective inhibitor of ER stress. Here, we investigated the role of TUDCA in the progression of tubular injury in DN. For eight weeks, being treated with TUDCA at 250 mg/kg intraperitoneal injection (i.p.) twice a day, diabetic db/db mice had significantly reduced blood glucose, albuminuria and attenuated renal histopathology. These changes were associated with a significant decreased expression of ER stress markers. At the same time, diabetic db/db mice had more TUNEL-positive nuclei in the renal tubule, which were attenuated by TUDCA treatment, along with decreases in ER stress–associated apoptotic markers in the kidneys. In summary, the effect of TUDCA on tubular injury, in part, is associated with inhibition of ER stress in the kidneys of diabetic db/db mice. TUDCA shows potential as a therapeutic target for the prevention and treatment of DN.

## 1. Introduction

Diabetic nephropathy (DN) remains the main cause of chronic kidney disease (CKD) and end-stage renal disease (ESRD) [1], accounting for nearly 50% of all cases of patients requiring dialysis each year in many industrialized countries [2]. Changes in the glomerulus play important roles in the pathophysiology of the diabetic kidney [3]. However, a growing body of evidence has shown that tubulointerstitial injury also might be an important hallmark of DN and a better predictor of renal disease progression than glomerular injury [4]. In addition, tubular cells are demonstrated to be key targets of hyperglycemia [5]. Several studies have validated that the apoptosis of tubular cells is frequently detected in renal sections from humans, mice and rats with diabetic mellitus (DM) [6,7,8], indicating that apoptosis plays an important role in the physiopathologic mechanism of tubular injury in the development of DN.

Endoplasmic reticulum (ER) stress has been considered to be a mediator of apoptosis [9,10]. A variety of insults [11] such as hypoxia and oxidative stress may disturb ER homeostasis, which can induce ER stress and the subsequent unfolded protein response (UPR). Glucose-regulated protein of 78 kDa (GRP78) serves as a central regulator of three main UPR sensors, namely activating transcription factor (ATF6), inositol-requiring enzyme (IRE)-1α, and protein kinase RNA-like ER kinase (PERK), which initiate the UPR signaling pathway under ER stress [12]. If the stress is too prolonged or severe and ER homeostasis cannot be restored, an apoptotic signaling pathway is triggered to ensure the survival of the organism as a last line of defense. Previous studies have demonstrated that ER stress has been well appreciated to contribute to the development and progression of chronic kidney diseases [13,14]. Furthermore, ER stress–induced apoptosis is involved in DN [15,16]. However, the relationship between ER stress and tubular injury, especially in diabetic nephropathy, has a lack of in-depth research.

Tauroursodeoxycholic acid (TUDCA) is an effective inhibitor of ER stress [17] which is shown to modify metabolic disorders in obese mice [18]. It has reported that TUDCA could protect streptozotocin (STZ)-induced diabetic retinopathy rats [19]. In addition, a recent study has suggested that some chemical inhibitors of ER stress may be helpful in diabetic glomerulopathy [20].

This study aims to investigate the effect of ER stress on tubular cell apoptosis, evaluate the effects of TUDCA treatment on ER stress and tubular injury in diabetic db/db mice and then search for a potential therapy for the treatment of DN.

## 2. Materials and Methods

### 2.1. Animals and Grouping

Male db/db (C57BLKS/J-LepRdb/LepRdb) mice at six weeks of age and age-matched lean non-diabetic littermates db/m (C57BLKS/J-LepRdb/+) mice were purchased from the National Mode Animal Centre of Nanjing University (Nanjing, China). After adaptive feeding for two weeks, db/db mice were randomly divided into two groups: the diabetic nephropathy group (DN; *n* = 10) and the TUDCA treatment group (DN+T; *n* = 10). Db/m mice were defined as the normal control group (NC; *n* = 10). TUDCA (Merck Millipore, Billerica, MA, USA) was administered by intraperitoneal injection (i.p.) twice a day for eight weeks to the DN + T group at a dose of 250 mg/kg [17]. The NC and DN group were administered the equal amounts of normal saline. All mice were housed in the specific pathogen–free (SPF) room and had free access to normal food and water. All animal experimental protocols were approved by the Laboratory Animals Ethical Committee of the Sixth People’s Hospital Affiliated to Shanghai Jiaotong University (ethical approval code No. 2016-0205).

### 2.2. Physical and Biochemical Analysis

Body weight and blood glucose were measured. The 24 h urine samples were collected in metabolic cages at the end of the 16 weeks. The urinary albumin and urinary creatinine concentration were assayed using mouse albumin ELISA Quantitation Set (Bethyl Laboratories, Montgomery, TX, USA) and a commercial ELISA kit (Cayman Chemical, Ann Arbor, MI, USA) according to the manufacturer’s instructions.

### 2.3. Histology Analysis

Formalin-fixed and paraffin-embedded renal tissues were sectioned (4 µm thickness) and stained with Periodic Acid-Schiff (PAS) and Masson Trichrome. To assess the degree of fibrosis, 10 non-overlapping fields of each section and eight slides per group were randomly chosen. Tubulointerstitial injury was graded as follows: grade 0, normal; grade 1, the area of interstitial inflammation and fibrosis, tubular atrophy, and dilation with cast formation involving <25% of the field; grade 2, lesion area between 25% and 50% of the field; and grade 3, lesion area >50% of the field. The indices for tubulointerstitial injury were calculated by averaging the grades assigned to all fields of tubules.

For immunohistochemistry, paraffin-embedded renal sections (4 μm thickness) were dewaxed and hydrated. Slides were boiled in 10 mM sodium citrate buffer (pH 6) for 10 min and cooled for 1 h at room temperature. After 10 min incubation in 0.3% hydrogen peroxide, sections were blocked with normal horse serum for 30 min at 37 °C, and then stained with primary antibodies (both from Cell Signaling Technology, Danvers, MA, USA; 1:100 with GRP78 and 1:50 with CCAAT/enhancer-binding protein homologous protein, CHOP) overnight at 4 °C. After washing with rinse buffer (DAKO, Glostrup, Denmark), sections were incubated with biotinylated anti-rabbit and anti-mouse IgG (Vector Laboratories, Burlingame, CA, USA), respectively, and visualized in brown using diaminobenzidine tetrahydrochloride solution as chromogen and hematoxylin as counterstain. All the measurements were detected by ImageProPlus Systems.

### 2.4. Terminal Deoxynucleotidyl Transferase (TdT)-Mediated dUTP Nick-End-Labeling (TUNEL) Assay

TUNEL staining using the DeadEnd™ Colometric TUNEL System (Promega, Madison, WI, USA) was carried out according to the manufacturer’s protocols. In brief, four-micrometer paraffin-embedded tissue sections were dewaxed and hydrated. Then sections were incubated with proteinase K (20 µg/mL) for 15 min at room temperature, blocked in 1.5% H_2_O_2_ for 10 min at 37 °C and treated with TUNEL reaction mixture. At least ten fields per slide and eight slides per group were scored for apoptotic nuclei. TUNEL-positive cells were counted under the light microscope by two independent pathologists in a blind fashion.

### 2.5. RNA Extraction and Real-Time PCR

Total RNA was extracted from renal cortex according to the manufacturer’s protocols for Trizol reagent (Invitrogen, Carlsbad, CA, USA) and the purity and concentration of RNAs were detected with spectrophotometer (Nanodrop2000). Total RNA (1000 ng) was reverse transcribed with SuperScript III Reverse Transcriptase kit (Invitrogen, Carlsbad, CA, USA). The cDNA was performed for quantitative real-time PCR analysis using a StepOnePlus System (Applied Biosystems, Foster City, CA, USA) with a SYBR^®^ Green PCR Kit (QIAGEN, GmbH, Hilden, Germany). The oligonucleotide primers for target genes were used as follows: GRP78: forward 5′-AGGCTAAGAGAGCCTTGTCT-3′ and reverse 5′-TCCAACACTTTCTGGACAGG-3′; CHOP: forward 5′-TTCACCTTGGAGACGGTG-3′ and reverse 5′-CGCAGGGTCAAGAGTAGTG-3′. All samples were analyzed in triplicate. The mRNA expression levels were normalized to those of GAPDH of the same cDNA sample. Relative quantification of gene expression was calculated using the 2^−ΔΔCt^ method.

### 2.6. Western Blot Analysis

Renal cortex were lysed in RIPA buffer containing phosphatase inhibitor cocktail with a sonicator and centrifuged at 14,000 rpm for 15 min at 4 °C. The protein concentration was determined with a Bio-Rad protein assay kit (Bio-Rad, Hercules, CA, USA; bovine serum albumin was used as a standard). Equal amounts (40 μg) of protein extracts were subjected to 10%–15% sodium dodecylsulfate-polyacrylamide gel electrophoresis (SDS-PAGE) and transferred to polyvinylidene fluoride membranes (Millipore). After being blocked with 3% bovine serum albumin (BSA) for 1 h at room temperature, the membranes were incubated with the following primary antibodies (all from Cell Signaling Technology, Danvers, MA, USA): anti-GRP78 (1:1000), anti-CHOP (1:1000), anti-cleaved caspase12 (1:1000), anti-cleaved caspase3 (1:1000) and anti-GAPDH (1:1000) antibodies overnight at 4 °C. Then the membranes were washed in Tris-buffered NaCl solution containing 0.1% Tween 20, and then incubated with horseradish peroxidase-conjugated secondary antibodies for 1 h at room temperature. Imaging was detected using the BIO-RAD Imaging System (Bio-Rad, Hercules, CA, USA) with chemiluminescence detection reagents (Thermo Fisher Scientific, Waltham, MA, USA). The densitometry of the bands was performed by image-scanning analysis software (UVP Inc. Upland, CA, USA) and described as the fold change from the NC group.

### 2.7. Statistical Analysis

Normally distributed data are presented as mean ± SEM, and one-way ANOVA followed by the Tukey’s Multiple Comparison Test was used to compare parametric data whereas categorical variables were presented as frequencies and Kruskal-Wallis test followed by the Mann-Whitney U test was used for nonparametric data comparison. *p* < 0.05 was considered as statistical significance. Statistical analyses were performed by SPSS 19.0 (IBM, Armonk, NY, USA).

## 3. Results

### 3.1. Effects of TUDCA on Biochemical Markers in the Different Groups

**Figure 1:**
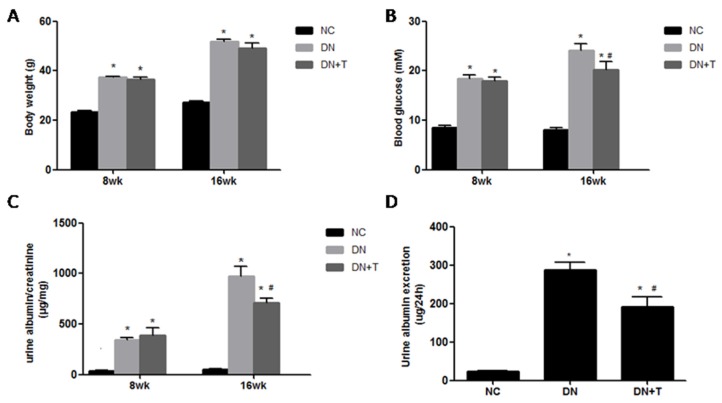
Physical and biochemical characteristics in various groups of mice. The DN + T group (db/db mice treated with TUDCA, *n* = 10) were administrated with 250 mg/kg TUDCA twice a day by i.p. injection for eight weeks. Mice in the NC group (normal control db/m mice, *n* = 10) or DN group (diabetic db/db mice, *n* = 10) received the equal amount of normal saline. Body weight (**A**); blood glucose (**B**); the ratio of urinary albumin and creatinine (**C**); and urine albumin excretion (**D**) of db/db mice were decreased by TUDCA treatment. Results are shown as means ±SEM. * *p* < 0.05 versus NC group. # *p* < 0.05 versus DN group.

As shown in Figure 1, all db/db mice displayed uniformly increased body weight (Figure 1A) levels compared with the corresponding db/m mice at eight weeks and 16 weeks of age. The diabetic db/db mice showed severe proteinuria compared to the non-diabetic db/m mice (*p* < 0.05). Treatment with TUDCA significantly reduced blood glucose (Figure 1B) and ameliorated proteinuria (Figure 1C,D) in diabetic mice (*p* < 0.05). These data also indicated that TUDCA could distinctly prevent the progression of DN.

### 3.2. TUDCA Improves the Renal Morphologyin db/db Mice

**Figure 2:**
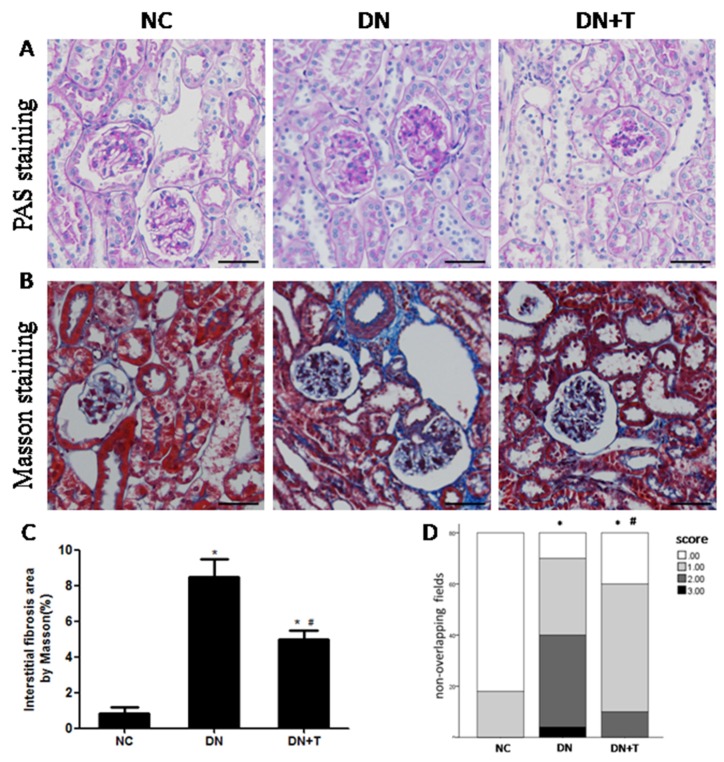
Histological analysis of kidney. Representative sections from kidneys of mice from each group at 16 weeks of age and stained with: (**A**) PAS (original magnification, 200×); and (**B**) Masson’s trichrome. Scale bar = 50 μm; Quantitive analysis of interstitial fibrosis area (**C**); and tubulointerstitial injury (**D**). Normally distributed data were expressed as means ± SEM whereas categorical variables were described as frequencies for 10 non-overlapping fields of each section and eight mice per group * *p* < 0.05 versus NC group. # *p* < 0.05 versus DN group.

PAS (Figure 2A) and Masson trichrome (Figure 2B) staining showed typical renal histopathological changes in db/db mice at 16 weeks, including mesangial cell proliferation, focal mesangial matrix expansion in glomeruli and collagen deposition in the tubulointerstitium compared to normal control db/m mice. However, these changes were significantly ameliorated by TUDCA treatment when compared with the untreated db/db mice. Furthermore, renal interstitial fibrosis (Figure 2C) was less severe and the tubulointerstitial injury score (Figure 2D) was lower in TUDCA-treated mice than those in untreated diabetic mice.

### 3.3. TUDCA Inhibits ER Stress Induced by Diabetes in the Kidneys of db/db Mice

**Figure 3:**
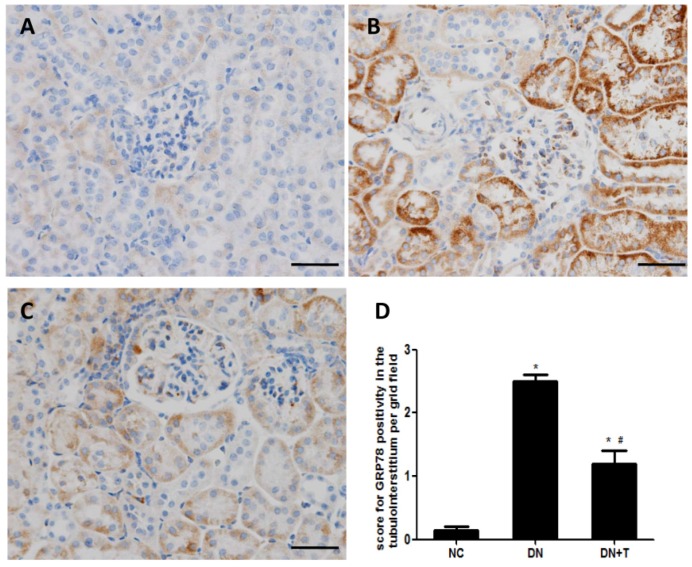
Effect of TUDCA on immune staining of GRP78 in the renal tissues of db/db mice. TUDCA treatment for eight weeks could attenuate diabetes-induced GRP78 expression. (**A**–**C**) Representative photographs of immune staining for GRP78 in the renal tissues of various groups of mice. (**A**) Non-diabetic db/m mice, NC group; (**B**) diabetic db/db mice, DN group; (**C**) diabetic db/db mice + TUDCA, DN + T group. Scale bar = 50 μm; (**D**) Quantitative analysis of GRP78 per field. Values are presented as means ± SEM for 10 non-overlapping fields of each section and eight mice per group. * *p* < 0.05 versus NC group. # *p* < 0.05 versus DN group.

**Figure 4:**
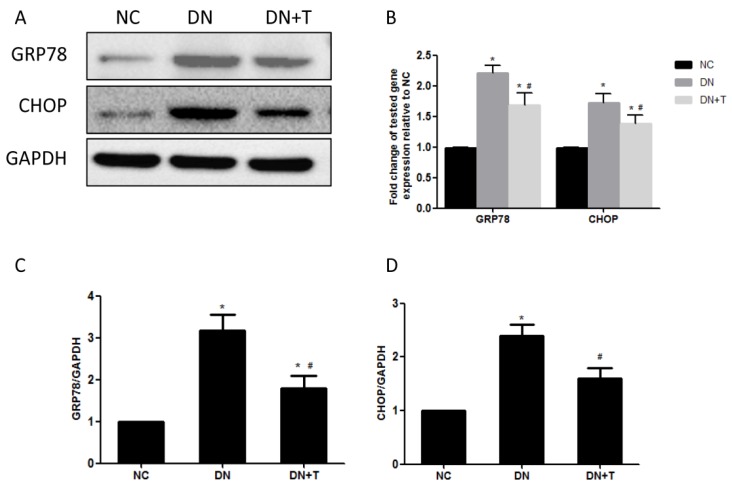
Effect of TUDCA on diabetes-induced ER stress in the renal cortex of db/db mice. TUDCA administration for eight weeks could decrease diabetes-induced ER stress markers. (**A**) The kidney tissue lysates were subjected to Western blot analysis with specific antibodies against ER stress markers (GRP78 and CHOP). Each sample was normalized to GAPDH expression; (**B**) Relative transcript levels of GRP78 and CHOP were detected by real-time PCR and normalized to expression of GAPDH. Densitometric analysis of Western blots for GRP78; (**C**) and CHOP; (**D**) protein in extracts from renal cortex. Values are presented as means ± SEM (*n* = 10). * *p* < 0.05 versus NC group. # *p* < 0.05 versus DN group.

To examine whether ER stress is induced in the kidneys of db/db mice, we measured the expression of ER stress markers in the kidneys of db/db mice. Immunohistochemistry analysis showed that GRP78 was predominantly expressed in the renal tubules and few stainings were detected in the glomeruli. Increased GRP78 staining was detected in the proximal renal tubules of diabetic db/db mice compared with control db/m mice, which could be significantly ameliorated by TUDCA treatment (Figure 3A–C). We also examined ER stress–associated proteins (GRP78 and CHOP) in the renal cortex of db/db mice using Western blot analysis (Figure 4A) and found the levels of these proteins were markedly higher than in non-diabetic db/m mice, which were consistent with their mRNA level as assessed by real-time PCR assay (Figure 4B). These results indicated that ER stress is induced in the kidneys of db/db mice, and TUDCA effectively attenuates the level of ER stress induced by diabetes.

### 3.4. TUDCA Inhibits ER Stress–Associated Apoptosis Pathways

**Figure 5:**
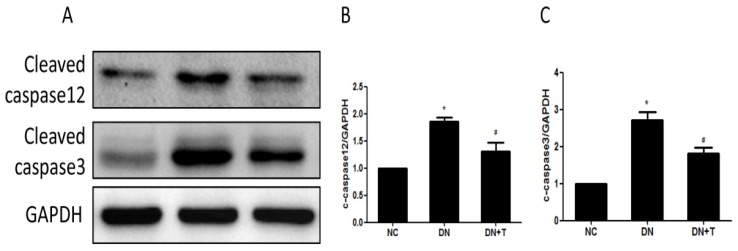
Effects of TUDCA on apoptosis in the kidney of diabetic mice. (**A**) Treating db/db mice with TUDCA for eight weeks could attenuate cleaved caspase12 and cleaved caspase3 protein expression as examined by Western blot. Each sample was normalized to GAPDH expression; Densitometric analysis of Western blots for cleaved caspase12 (**B**); and cleaved caspase3 (**C**) protein in extracts from renal cortex. Values are presented as means ± SEM (*n* = 10). * *p* < 0.05 versus NC group. # *p* < 0.05 versus DN group.

To further assess the nephroprotective effects of TUDCA on diabetic db/db mice, the apoptosis-related protein expression levels were determined by Western blot. As shown in Figure 5A, cleaved caspase12 and cleaved caspase3 were markedly increased in db/db mice compared with the db/m mice, and significantly attenuated by TUDCA treatment, indicating ER stress–induced apoptosis was alleviated by TUDCA treatment.

### 3.5. TUDCA Reduces the Apoptosis of Tubular Cell in db/db Mice

**Figure 6:**
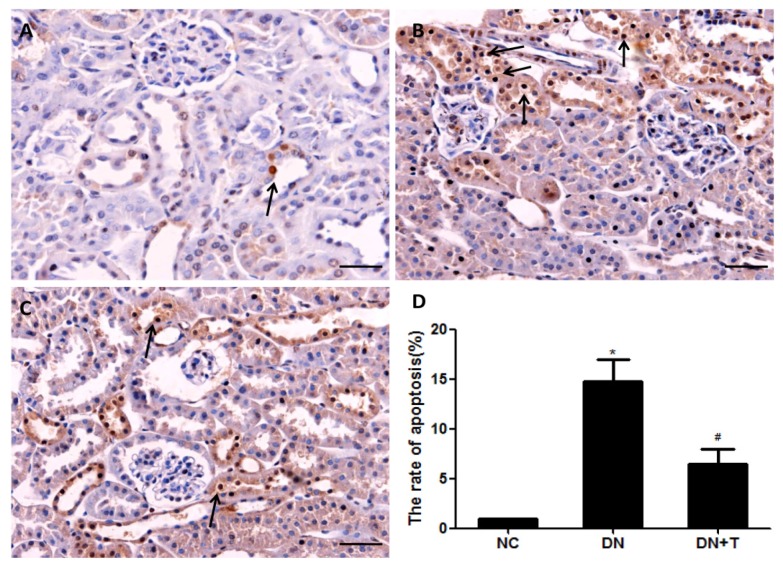
Effect of TUDCA on apoptotic tubular cells in renal tissues of db/db mice treated with TUDCA for eight consecutive weeks. Apoptosis of tubular cells in kidneys of each group: (**A**) non-diabetic db/m mice, NC group; (**B**) diabetic db/db mice, DN group; (**C**) diabetic db/db mice + TUDCA, DN+T group, were determined by TUNEL assay. Scale bar = 50 μm. TUNEL-positive cells in renal tubules were presented by arrows; (**D**) Quantitative analysis of TUNEL-positive stained tubular cells. Values are presented as means ± SEM for 10 non-overlapping fields of each section and eight mice per group. * *p* < 0.05 versus NC group. # *p* < 0.05 versus DN group.

To assess tubular cell apoptosis in the diabetic kidney, the renal tissue sections were performed with an in situ TUNEL assay. At 16 weeks, the diabetic db/db mice showed increased apoptotic tubular cells when compared with the non-diabetic db/m mice (Figure 6); the db/db mice treated with TUDCA displayed decreased apoptotic tubular cells compared with DN group. These data demonstrated that TUDCA treatment could attenuate the ER stress–induced tubular cell apoptosis in type 2 diabetic kidneys.

## 4. Discussion

In the present study, we demonstrated that the ER stress signaling pathway was activated in the kidneys of db/db mice, which proved ER stress is involved in the pathogenesis of diabetic nephropathy. Treating with TUDCA, a small molecular chemical chaperon, which effectively inhibited ER stress, could lower the blood glucose level, alleviate tubulointerstitial fibrosis, improve diabetic nephropathy–associated parameters, including the urine albumin and creatinine ratio and urine albumin excretion, and reduce apoptotic tubular cells in diabetic db/db mice.

Diabetic nephropathy is characterized by glomerulosclerosis, tubular atrophy and tubulointerstitial fibrosis (TIF). Although glomerular lesions are a focus of renal injury in diabetes [21], growing evidence demonstrated the key role of tubule injury in the progression of the disease [22]. The apoptosis of tubular cells has been taken into account as a primary cause of tubular atrophy and tubulointerstitial fibrosis [23]. ER stress is recognized to be involved in the pathogenesis of the apoptosis of tubule epithelial cells [24]. Thus, targeting the inhibition of ER stress–induced tubular cell apoptosis may provide new therapeutic approaches for DN.

ER stress is considered as a mediator in the homeostasis of ER and a protective response mechanism of cells. Normally, the adaptive response of ER can restore ER function and protect cells through upregulating ER-resident chaperons, such as GRP78. GRP78 is a critical molecular chaperon of ER, which binds to three key proteins (PERK, IRE1 and ATF6) located at the ER membrane to form a complex. When unfolded or misfolded proteins accumulate in the ER, GRP78 is released from the complex and is upregulated through the UPR to induce ER stress [25]. In the renal sections of mice, immunohistochemical results showed that GRP78 staining was increased in the renal proximal tubule of db/db mice, which was consistent with the results of the Western blot assay. These data indicated ER stress is induced in renal tubules of diabetic mice.

If ER homeostasis cannot be restored, ER-associated apoptotic signaling pathways can be initiated [25]. CHOP is considered a transcriptionally regulated gene which is involved in the ER stress–induced apoptotic pathway and acts as a proapoptotic protein [26]. It was reported that the expression of CHOP was increased during ER stress and might play an important role in mediating the onset of ER stress–associated apoptosis [27]. CHOP knockout in diabetic mice protected the kidney against injury induced by ER stress [28]. Caspase12 is exclusively located at the cytoplasmic side of ER in rodents [29,30], which is cleaved and activated during the ER stress–induced apoptosis cascade. Then it can further trigger downstream caspase 3 in the cytosol [31]. Therefore, the levels of CHOP and caspase-12 can reflect whether the ER stress–induced apoptosis pathway is activated. The TUNEL assay demonstrated that the apoptotic tubular cells increased in diabetic db/db mice compared with the control db/m mice, which indicated ER stress–induced apoptosis was triggered in the kidneys of the diabetic db/db mice.

In the past few decades, chemical chaperones such as 4-Phenylbutyrate (4-PBA) and TUDCA were demonstrated to repress ER stress by facilitating protein folding [32,33]. The 4-PBA attenuated tunicamycin-induced acute kidney injury via repressing CHOP [34]. Like 4-PBA, TUDCA also is an inhibitor of ER stress, which alleviated ischemia-/reperfusion-induced acute kidney injury by inhibiting ER stress [35] and protected kidney epithelial cell injury against albuminuria in STZ-induced diabetic mice [36]. Furthermore, TUDCA prevented cultured mouse podocytes from apoptosis induced by advanced glycation end products via blocking an ER stress–mediated apoptotic pathway [37].

In this study, diabetic nephropathy–associated parameters and renal histopathology were improved, along with the downregulation of ER stress markers and inactivation of the ER stress–associated apoptotic pathway in TUDCA-injected db/db mice. These results indicated that the nephroprotective effects of TUDCA on db/db mice might be mediated, at least in part, via inhibiting ER stress. However, TUDCA was reported to ameliorate insulin resistance and restore glucose tolerance in mice with type 2 diabetes [17], and our data also showed that treating them with TUDCA could reduce blood glucose in diabetic db/db mice. Thus, as an ER stress inhibitor, the nephroprotective effects of TUDCA on db/db mice may be the indirect effects of its reduction of blood glucose. Whether the protective effect of TUDCA on the apoptosis of renal tubules is independent of blood glucose reduction still needs to be further studied in the future.

## 5. Conclusions

In summary, we demonstrated in vivo that ER stress seemed to play a critical role in diabetic db/db mice. The inhibition of ER stress via TUDCA not only could lower blood glucose and reduce albuminuria, as well as improve renal histopathology, but it could also attenuate increased apoptosis of tubules in diabetic db/db mice, which might be associated with reduced endoplasmic reticulum stress. These data also provide further evidence for the application of TUDCA in the prevention of diabetic nephrology.

## References

[1] Collins A.J., Foley R.N., Herzog C., Chavers B., Gilbertson D., Herzog C., Ishani A., Johansen K., Kasiske B., Kutner N. (2013). Us renal data system 2012 annual data report. Am. J. Kidney Dis..

[2] Atkins R.C., Zimmet P. (2010). Diabetic kidney disease: Act now or pay later. Nephrol. Dial. Transplant..

[3] Kanwar Y.S., Sun L., Xie P., Liu F.Y., Chen S. (2011). A glimpse of various pathogenetic mechanisms of diabetic nephropathy. Annu. Rev. Pathol..

[4] Gilbert R.E., Cooper M.E. (1999). The tubulointerstitium in progressive diabetic kidney disease: More than an aftermath of glomerular injury?. Kidney Int..

[5] Jenkin K.A., McAinch A.J., Zhang Y., Kelly D.J., Hryciw D.H. (2015). Elevated cannabinoid receptor 1 and G protein-coupled receptor 55 expression in proximal tubule cells and whole kidney exposed to diabetic conditions. Clin. Exp. Pharmacol. Physiol..

[6] Brezniceanu M.L., Lau C.J., Godin N., Chenier I., Duclos A., Ethier J., Filep J.G., Ingelfinger J.R., Zhang S.L., Chan J.S. (2010). Reactive oxygen species promote caspase-12 expression and tubular apoptosis in diabetic nephropathy. J. Am. Soc. Nephrol..

[7] Brezniceanu M.L., Liu F., Wei C.C., Chenier I., Godin N., Zhang S.L., Filep J.G., Ingelfinger J.R., Chan J.S. (2008). Attenuation of interstitial fibrosis and tubular apoptosis in db/db transgenic mice overexpressing catalase in renal proximal tubular cells. Diabetes.

[8] Kumar D., Robertson S., Burns K.D. (2004). Evidence of apoptosis in human diabetic kidney. Mol. Cell. Biochem..

[9] Pallepati P., Averill-Bates D.A. (2011). Activation of ER stress and apoptosis by hydrogen peroxide in hela cells: Protective role of mild heat preconditioning at 40 degrees C. Biochim. Biophys. Acta.

[10] Wang X., Olberding K.E., White C., Li C. (2011). Bcl-2 proteins regulate er membrane permeability to luminal proteins during ER stress-induced apoptosis. Cell Death Differ..

[11] Yoshida H. (2007). ER stress and diseases. FEBS J..

[12] Cybulsky A.V. (2013). The intersecting roles of endoplasmic reticulum stress, ubiquitin- proteasome system, and autophagy in the pathogenesis of proteinuric kidney disease. Kidney Int..

[13] Taniguchi M., Yoshida H. (2015). Endoplasmic reticulum stress in kidney function and disease. Curr. Opin. Nephrol. Hypertens..

[14] Fan Y., Xiao W., Li Z., Li X., Chuang P.Y., Jim B., Zhang W., Wei C., Wang N., Jia W. (2015). RTN1 mediates progression of kidney disease by inducing ER stress. Nat. Commun..

[15] Baban B., Liu J.Y., Mozaffari M.S. (2013). Endoplasmic reticulum stress response and inflammatory cytokines in type 2 diabetic nephropathy: Role of indoleamine 2,3-dioxygenase and programmed death-1. Exp. Mol. Pathol..

[16] Inagi R. (2010). Endoplasmic reticulum stress as a progression factor for kidney injury. Curr. Opin. Pharmacol..

[17] Ozcan U., Yilmaz E., Ozcan L., Furuhashi M., Vaillancourt E., Smith R.O., Gorgun C.Z., Hotamisligil G.S. (2006). Chemical chaperones reduce ER stress and restore glucose homeostasis in a mouse model of type 2 diabetes. Science.

[18] Chen Y., Wu Z., Zhao S., Xiang R. (2016). Chemical chaperones reduce ER stress and adipose tissue inflammation in high fat diet-induced mouse model of obesity. Sci. Rep..

[19] Wang C.F., Yuan J.R., Qin D., Gu J.F., Zhao B.J., Zhang L., Zhao D., Chen J., Hou X.F., Yang N. (2016). Protection of tauroursodeoxycholic acid on high glucose-induced human retinal microvascular endothelial cells dysfunction and streptozotocin-induced diabetic retinopathy rats. J. Ethnopharmacol..

[20] Cao A.L., Wang L., Chen X., Wang Y.M., Guo H.J., Chu S., Liu C., Zhang X.M., Peng W. (2016). Ursodeoxycholic acid and 4-phenylbutyrate prevent endoplasmic reticulum stress-induced podocyte apoptosis in diabetic nephropathy. Lab. Investig..

[21] Drummond K., Mauer M., International Diabetic Nephropathy Study Group (2002). The early natural history of nephropathy in type 1 diabetes: II. Early renal structural changes in type 1 diabetes. Diabetes.

[22] Sun H.L., Sun L., Li Y.Y., Shao M.M., Cheng X.Y., Ge N., Lu J.D., Li S.M. (2009). Ace-inhibitor suppresses the apoptosis induced by endoplasmic reticulum stress in renal tubular in experimental diabetic rats. Exp. Clin. Endocrinol. Diabetes.

[23] Docherty N.G., O’Sullivan O.E., Healy D.A., Fitzpatrick J.M., Watson R.W. (2006). Evidence that inhibition of tubular cell apoptosis protects against renal damage and development of fibrosis following ureteric obstruction. Am. J. Phys. Ren. Physiol..

[24] Asmellash S., Stevens J.L., Ichimura T. (2005). Modulating the endoplasmic reticulum stress response with trans-4,5-dihydroxy-1,2-dithiane prevents chemically induced renal injuryin vivo. Toxicol. Sci..

[25] Schroder M., Kaufman R.J. (2005). ER stress and the unfolded protein response. Mutat. Res..

[26] Marciniak S.J., Yun C.Y., Oyadomari S., Novoa I., Zhang Y., Jungreis R., Nagata K., Harding H.P., Ron D. (2004). Chop induces death by promoting protein synthesis and oxidation in the stressed endoplasmic reticulum. Genes Dev..

[27] Jing G., Wang J.J., Zhang S.X. (2012). ER stress and apoptosis: A new mechanism for retinal cell death. Exp. Diabetes Res..

[28] Wu J., Zhang R., Torreggiani M., Ting A., Xiong H., Striker G.E., Vlassara H., Zheng F. (2010). Induction of diabetes in aged C57B6 mice results in severe nephropathy: An association with oxidative stress, endoplasmic reticulum stress, and inflammation. Am. J. Pathol..

[29] Nakagawa T., Yuan J. (2000). Cross-talk between two cysteine protease families activation of caspase-12 by calpain in apoptosis. J. Cell Biol..

[30] Nakagawa T., Zhu H., Morishima N., Li E., Xu J., Yankner B.A., Yuan J. (2000). Caspase-12 mediates endoplasmic-reticulum-specific apoptosis and cytotoxicity by amyloid-beta. Nature.

[31] Hitomi J., Katayama T., Taniguchi M., Honda A., Imaizumi K., Tohyama M. (2004). Apoptosis induced by endoplasmic reticulum stress depends on activation of caspase-3 via caspase-12. Neurosci. Lett..

[32] Burrows J.A., Willis L.K., Perlmutter D.H. (2000). Chemical chaperones mediate increased secretion of mutant alpha 1-antitrypsin (alpha 1-AT) Z: A potential pharmacological strategy for prevention of liver injury and emphysema in alpha 1-AT deficiency. Proc. Natl. Acad. Sci. USA.

[33] Ozcan L., Ergin A.S., Lu A., Chung J., Sarkar S., Nie D., Myers M.G., Ozcan U. (2009). Endoplasmic reticulum stress plays a central role in development of leptin resistance. Cell Metab..

[34] Carlisle R.E., Brimble E., Werner K.E., Cruz G.L., Ask K., Ingram A.J., Dickhout J.G. (2014). 4-phenylbutyrate inhibits tunicamycin-induced acute kidney injury via chop/gadd153 repression. PLoS ONE.

[35] Gao X., Fu L., Xiao M., Xu C., Sun L., Zhang T., Zheng F., Mei C. (2012). The nephroprotective effect of tauroursodeoxycholic acid on ischaemia/reperfusion-induced acute kidney injury by inhibiting endoplasmic reticulum stress. Basic Clin. Pharmacol. Toxicol..

[36] Fang L., Xie D., Wu X., Cao H., Su W., Yang J. (2013). Involvement of endoplasmic reticulum stress in albuminuria induced inflammasome activation in renal proximal tubular cells. PLoS ONE.

[37] Chen Y., Liu C.P., Xu K.F., Mao X.D., Lu Y.B., Fang L., Yang J.W., Liu C. (2008). Effect of taurine-conjugated ursodeoxycholic acid on endoplasmic reticulum stress and apoptosis induced by advanced glycation end products in cultured mouse podocytes. Am. J. Nephrol..

